# Functional Anatomy of the Pharynx of *Glycera tridactyla* Schmarda, 1861 (Annelida: Glyceriformia: Glyceridae)

**DOI:** 10.1002/jmor.70139

**Published:** 2026-06-05

**Authors:** Kleoniki Keklikoglou, Joachim Langeneck, Desirèe Dimichele, Emmanouela Vernadou, Eva Chatzinikolaou, Luigi Musco

**Affiliations:** ^1^ Institute of Marine Biology, Biotechnology and Aquaculture (IMBBC), Hellenic Centre for Marine Research (HCMR) Heraklion Crete Greece; ^2^ National Interuniversity Consortium for Marine Sciences (CoNISMa) Rome Italy; ^3^ Department of Biological and Environmental Sciences and Technologies (DiSTeBA) University of Salento Lecce Italy; ^4^ Earth and Marine Sciences Department (DiSTeM) University of Palermo Palermo Italy; ^5^ National Biodiversity Future Centre (NBFC) Palermo Italy

**Keywords:** annelida, micro‐CT, morphology, polychaeta, proboscis

## Abstract

The pharyngeal muscular system of polychaetes is highly complex and shows remarkable variation in structure and function among taxa. In this study, the functional anatomy of the pharynx of *G. tridactyla* was investigated using micro‐computed tomography (micro‐CT). Sixteen *Glycera* specimens were imaged in different pharyngeal positions to examine the roles of key muscular structures in pharyngeal movement. The high‐resolution imaging provided by micro‐CT allowed visualisation of the pharyngeal muscles in their original topography, revealing essential components for pharyngeal function, including the ring muscle, retensor muscles, intestinal retractor muscles, and longitudinal muscles of the buccal tube and oesophagus. Comparison with previous studies addressed gaps in our knowledge of Glyceridae functional anatomy. Specifically, detailed analysis of the muscular system in different pharyngeal positions clarified the mechanisms of proboscis movement, which indicate the rapid and effective responses of these worms during burrowing and feeding behaviour.

## Graphical abstract

Micro‐CT technology was used to investigate the functional anatomy of the pharynx in the polychaete *Glycera tridactyla*. The study revealed a complex system of pharyngeal muscles, including ring muscles, retensor muscles, intestinal retractor muscles, and longitudinal muscles of the buccal tube and oesophagus. This muscular system plays a crucial role in proboscis movement, enabling efficient burrowing and feeding behaviour in these polychaetes.

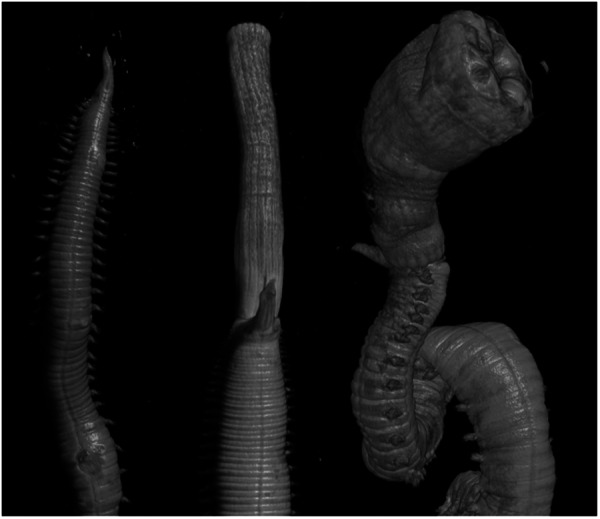

## 1 Introduction

Studying the morphology of the muscular system in Annelida can offer valuable insights into the phylogenetic relationships and the direction of evolutionary changes in this taxonomic group (Purschke 2006). Furthermore, the description of the muscular system of polychaetes is essential for the interpretation of their behaviour, lifestyle and ecological role (Tzetlin and Filippova 2005). The general structure of a polychaete muscular system is overall well known and is composed of multiple elements, including circular and longitudinal body‐wall fibres, as well as parapodial, chaetal, oblique, diagonal, and dorsoventral muscles, along with the muscular components of the septa and mesenteries (Tzetlin and Filippova 2005). These elements are usually arranged in a modular, repeated way, and remain virtually identical along all the metamers structuring the body of the polychaete. However, muscles associated with non‐metameric parts of the polychaete anatomy might significantly differ among different polychaete groups, and are comparatively less studied (Purschke 1988; Bergter et al. 2008).

Previous anatomical/morphological studies mainly relied on traditional techniques such as dissection and histology, occasionally complemented by more advanced approaches, including transmission electron microscopy and confocal laser scanning microscopy (Purschke 2006). Micro‐computed tomography (micro‐CT), a non‐destructive three‐dimensional imaging technique, has been recently applied in taxonomic and anatomical studies of polychaetes (e.g., Faulwetter et al. 2013, 2014; Parapar et al. 2018, 2019, 2025). Due to its ability to visualise internal structures in their original topography (Descamps et al. 2014), micro‐CT has also been used in functional anatomical studies (e.g., Nickel et al. 2006; Dinley et al. 2010; Castejón et al. 2018; Evans and Elder 2024). However, in polychaetes, only a limited number of studies have addressed the relationship between anatomy and function (e.g., Dinley et al. 2010).

Among the various muscular systems in polychaetes, the pharyngeal organ exhibits remarkable structural and functional diversity across different taxa. The pharynx is described as the buccal organ and, in the case of an eversible pharynx, as a proboscis (Rouse et al. 2022). Based on its complexity and orientation, the pharyngeal organ can be categorised into four categories, namely simple axial proboscis, simple ventral buccal organ, ventral muscular proboscis, and muscular axial proboscis (Dales 1962; Purschke and Tzetlin 1996); in addition, some extreme adaptations led to the anatomical or functional loss of the buccal organ (Rouse et al. 2022). Within each category, substantial morphological diversity can be observed, particularly in taxa possessing a muscular proboscis. Muscular proboscis is typical of errant polychaetes, including both Errantia as redefined by Struck et al. (2011) and the early‐branching Amphinomida, suggesting an ancient origin of this type of pharynx. Among errant polychaetes, Amphinomida and Eunicida are characterised by a ventral muscular proboscis, while the order Phyllodocida is characterised by an axial muscular proboscis. Within the order Phyllodocida, the pharynx exhibits different structure and adaptations depending on the phylogenetic affinities and evolutionary pathways of organisms of the taxa involved. Specifically, depending on the taxon, the eversible pharynx can be restricted to a short, anterior part of the body (as in the families Nereididae and Nephtyidae) or occupy a sizable part of the body (as in Phyllodocidae and in the suborder Glyceriformia). In several families, the eversible pharynx is associated with jaws, mobile, non‐deformable structures based on collagen, in some cases with an additional mineralised layer, which are usually used to manipulate food items. When present, jaws occur in paired, mobile structures, in number of two (e.g., Nereididae, some Hesionidae), four (e.g., Aphroditiformia, Glyceridae), or multiple pieces (e.g., Goniadidae). The structure and shape of jaws change among families within the same order but in all cases they are embedded in a soft structure including both muscular and glandular components (Böggemann 2005, 2006; Rouse et al. 2022; Gonçalves et al. 2023).

Although some features of the buccal region (e.g., jaws, paragnaths, pharyngeal papillae) have been employed as taxonomic characters, relatively few studies focused on their functional anatomy (e.g., Wells 1937; Dales 1962; Uyeno and Kier 2015; Rodrigo et al. 2018). In this context, members of the family Glyceridae are particularly interesting due to the peculiar adaptations of their pharyngeal structures, which, based on fossil evidence, have remained essentially unchanged since the Carboniferous period, justifying their definition as living fossils (Böggemann 2006). Glyceridae are medium to large‐sized polychaetes, characterised by generally high motility and typically associated with soft sediments, where they are able to build complex burrows (Ockelmann and Vahl 1970), although some species also occur on hard substrates. In this family, the pharynx can occupy up to one‐third of the body length when everted and is primarily used to capture prey. Although the available gut content data are somewhat inconsistent, suggesting detritus feeding in some cases and active predation in others (Klawe and Dickie 1957; Fauchald and Jumars 1979), the majority of studies based on isotopic signatures, together with the presence of robust jaws coupled with venom glands, indicate that active predation is the predominant feeding mode in this family (Jumars et al. 2015). Predation in Glyceridae remains poorly understood, but species of the genus *Glycera* are known to feed on peracarid crustaceans and polychaete annelids (Jumars et al. 2015). In turn, *Glycera* species constitute a common food source for larger marine invertebrates, fish, and seabirds, particularly waders, making them an important component of coastal trophic networks (Klawe and Dickie 1957). In addition, the largest species are commonly harvested and sold as live bait (Klawe and Dickie 1957; Saito et al. 2014).

The general anatomy of Glyceridae pharynx has been explored in some detail, as it includes elements considered important for species identification, particularly with reference to proboscidial papillae and jaws (Böggemann 2002). The pharynx is characterised by several longitudinal nerves, with transverse thinner branches associated with pharyngeal papillae, including both mucous/serous secreting elements and mechanoceptors, which overall suggest a double sensorial and secreting role. The terminal bulb of the pharynx hosts two pairs of jaws, formed by a curved jaw piece, associated with a venom gland, and an accessory jaw piece, called “aileron”, involved in the movement of the jaw, and supporting its action during predation (Böggemann 2002). The shape and structure of the jaws are rather conserved in the family Glyceridae, while the shape of papillae and ailerons is often considered a diagnostic character and allows for the distinction between species of Glyceridae. In addition, different species seem to be able to produce different toxins with a different action mechanism, and the structure of venom glands and other pharyngeal internal structures seem to be highly species‐specific; however, the actual taxonomic informativeness of venom‐associated elements has not yet been fully explored (Böggemann 2002).

While the taxonomic relevance of pharyngeal structures in Glyceridae is well known ever since the beginning of the XX Century (Fauvel 1923), the functional anatomy of their pharynx is much less studied. The only studies exploring the general structure and suggesting the functioning of the eversion mechanism in Glyceridae are those by Wells (1937) and Dales (1962), who reconstructed it by dissecting individuals of *Glycera dibranchiata* Ehlers, 1868 and *Glycera convoluta* Keferstein, 1862 (= *Glycera tridactyla* Schmarda, 1861), respectively. By profiting of new non‐destructive technologies allowing in deep and precise anatomical characterisation, this study aims to increase knowledge about the functional anatomy of the pharynx of *G. tridactyla* by reconstructing it through micro‐CT, and comparing it with previous data provided by Wells (1937) and Dales (1962) on species belonging to the same genus.

## 2 Material and Methods

Sixteen individuals of *G. tridactyla* were sampled on April 4th 2024 in sandy sediments of La Strea Inlet, Porto Cesareo, Ionian Sea, Italy (40.2427° N; 17.9096° E) at a depth of approximately 0.5 m. In order to obtain different pharynx positions, 13 of the individuals were anesthetised with thymol in seawater to induce full or partial pharynx eversion, while 3 other individuals were fixed without anesthetisation to prevent pharynx eversion. All individuals were fixed in 4% formalin buffered with CaCO_3_ 0.03 M and transferred to 70% ethanol after at least 2 weeks to ensure proper fixation of the soft tissue and avoid deformations.

The morphology and anatomy of the pharynx of *G. tridactyla* were characterised through micro‐CT scans. Initially, specimens were scanned without any contrast agent to visualise the jaws. Subsequently, they were embedded in 0.3% phosphotungstic acid (PTA) dissolved in 70% ethanol (Metscher 2009) for 8 days to visualise the pharyngeal anatomy. Scanning was performed using a SkyScan 1172 microtomograph (Bruker, Kontich, Belgium) at the Hellenic Centre for Marine Research (HCMR). All specimens were scanned at 60 kV and 167 μA, without a filter, over a full 360° rotation. Images were acquired at a pixel size of approximately 4.5 μm with a camera binning of 1×1. The exposure time was 315–320 ms, with frame averaging set to 3. Projection images were reconstructed into cross‐sectional images using SkyScan's NRecon v.1.7.4.2 software (Bruker, Kontich, Belgium), with attenuation coefficients ranging from 0 to 0.2 for the unstained and 0 to 0.6 for the stained specimens. Volume renderings of each specimen were generated using CTVox v.3.3.0 r1403 software (Bruker, Kontich, Belgium).

In addition, dissections of the same specimens were performed under a stereomicroscope to validate the interpretation of anatomical structures observed in the micro‐CT images.

## 3 Results

**Figure 1:**
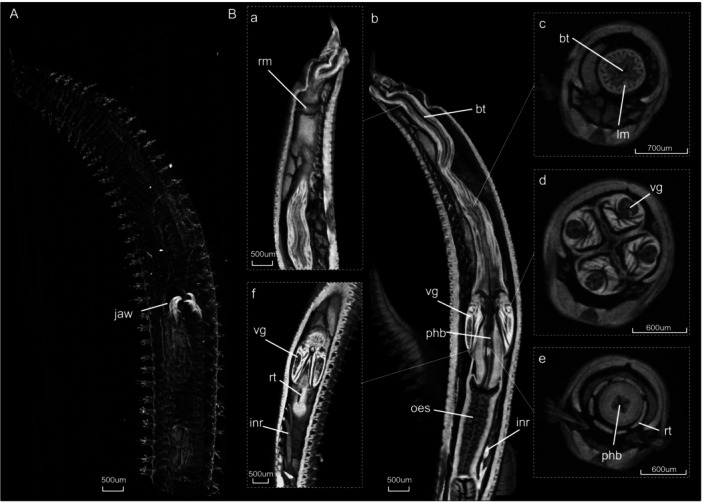
Anatomy of the (A) unstained and (B) stained *G. tridactyla* specimen with a retracted pharynx acquired through micro‐CT. In panel (B), (a,b,f) show volume renderings of the specimen, while (c–e) present cross‐sectional images. bt= buccal tube; inr = intestinal retractors; lm = longitudinal muscles of the buccal tube; oes = oesophagus; phb = pharyngeal bulb; rm = ring muscle; rt = retensors; vg = venom glands.

The anterior digestive system of *G. tridactyla* consists of a long, muscular buccal tube located posterior to the mouth, which extends to the pharyngeal bulb (Figure 1 Bb). In the anterior part of the pharyngeal bulb, four jaws (Figure 1A) are situated within four cushion‐like swellings, each containing associated muscles (Figure 1Bd), while the venom glands extend at the base of the jaws (Figure 1Bb,d,f). In the posterior region, six longitudinal retensor muscles run along the pharyngeal bulb (Figure 1Be,f). Beyond this structure, the digestive tract continues into the oesophagus and the intestine, the latter being connected to the body wall by a series of long and thick intestinal retractor muscles (Figure 1Bb,f).

**Figure 2:**
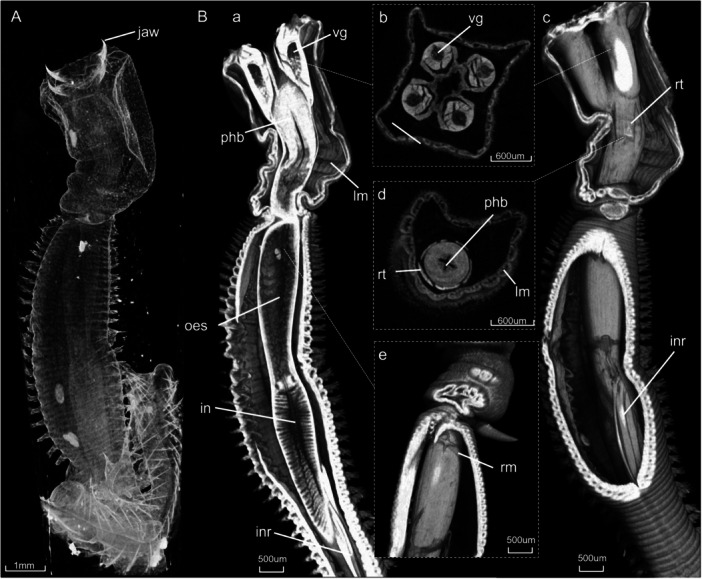
Anatomy of the (A) unstained and (B) stained *G. tridactyla* specimen with a protruded pharynx acquired through micro‐CT. In panel (B), (a,c,e) show volume renderings of the specimen, while (b,d) present cross‐sectional images. in = intestine, inr = intestinal retractors, lm= longitudinal muscles of the buccal tube, oes = oesophagus, phb = pharyngeal bulb, rm = ring muscle, rt = retensors, vg = venom glands.

**Figure 3:**
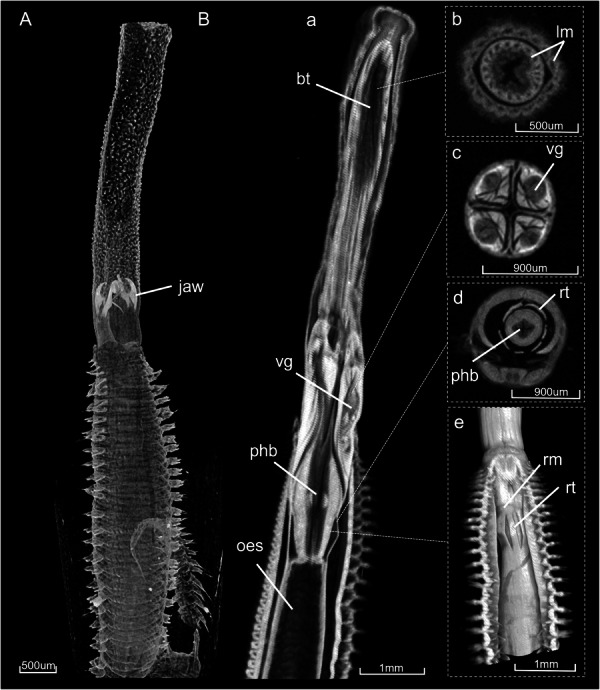
Anatomy of the (A) unstained and (B) stained *G. tridactyla* specimen with a partial protruded pharynx acquired through micro‐CT. In panel (B), (a,e) show volume renderings of the specimen, while (b‐d) present cross‐sectional images. bt= buccal tube, lm=longitudinal muscles of the buccal tube, oes = oesophagus, phb = pharyngeal bulb, rm = ring muscle, rt = retensors, vg = venom glands.

Micro‐CT images revealed several muscles important for the protrusion and retraction movements of the pharynx (Figures 1, 2, 3). During protrusion, the buccal tube represents the part of the proboscis that is everted during pharyngeal extrusion (Figure 2). The ring muscle, located around the 7th chaetiger in the anterior part of the buccal tube (Figure 2Be), contracts, and together with the coelomic hydrostatic pressure, the pharynx is protruded. In full protrusion, the pharynx moves forward and the jaws become fully exposed (Figure 2A). The muscles within the four cushion‐like swellings also appear to expand during this process (Figure 2Bb). Regarding the retensor muscles that cover the pharyngeal bulb, the micro‐CT images revealed that their length changes during retraction (Figure 1e,f) and protrusion (Figure 2Bc,d). Specifically, these muscles are contracted during retraction and relaxed during protrusion.

Partial pharyngeal protrusion may occur when the buccal tube is only partially everted, and the jaws are not exposed (Figure 3A). In this position, the ring muscle is partially contracted in order to stabilise a portion of the proboscis (Figure 3Be). The longitudinal muscles of the buccal tube and the oesophagus also play a role in controlling the partial protrusion (Figure 3Bb).

During pharyngeal retraction (Figure 1), the longitudinal muscles of the buccal tube and oesophagus are contracted (Figure 1Bc), however, the relaxation of the ring muscle is what leads to full retraction of the pharynx (Figure 1Ba). Furthermore, according to the micro‐CT images, when the pharynx is fully retracted, the intestinal retractor muscles are contracted and extend from the anterior part of the intestine, with insertion around the 36th chaetiger to the body wall (Figure 1Bb,f). In the fully protruded position, as the entire digestive system moves forward, these muscles become relaxed, and the position of their insertion in the intestine can be located around the 20th chaetiger, changing their orientation downwards (Figure 2Ba,c). The retensor muscles are also contracted during retraction, compacting the pharyngeal bulb (Figure 1Be,f).

**Figure 4:**
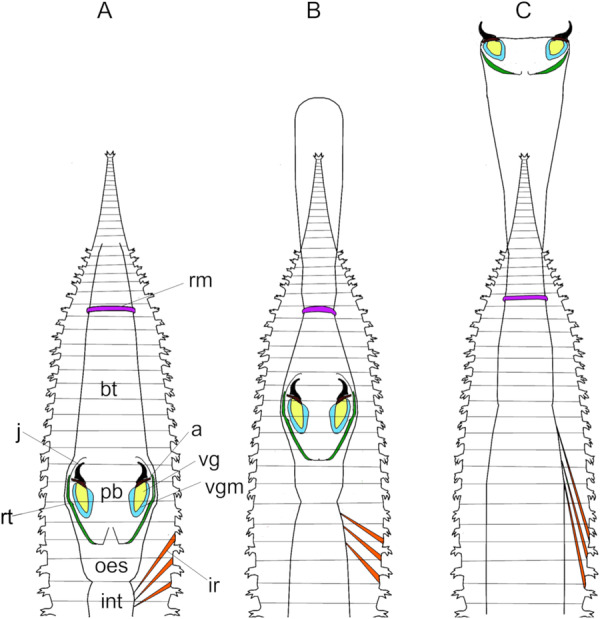
Schematic representation of the pharynx of *Glycera tridactyla* in three different configurations. (A) pharynx inverted (resting position); (B) pharynx partially everted; (C) pharynx completely everted. a= aileron, bt= buccal tube, int= intestine, ir= intestinal retractor muscle, j= jaw, oes= oesophagus, pb= pharyngeal bulb, rm= ring muscle, rt= retensor muscle, vg= venom gland, vgm= venom gland muscle. The number of intestinal retractors is higher than those displayed in the schematic drawings.

Based on the presented micro‐CT images, the primary muscles involved in pharyngeal movements include the ring muscle, the retensor muscles, the longitudinal muscles of the buccal tube and oesophagus, and the intestinal retractors. A schematic graphical representation of the pharyngeal movements is presented in Figure 4 showing the pharynx retracted (Figure 4A), partially protruded (Figure 4B) and fully protruded (Figure 4C).

## 4 Discussion

In this study, micro‐CT data revealed the basic anatomy of the anterior digestive system of *G. tridactyla* and the presence of a complex musculature associated with the digestive system, reflecting the functional capabilities of the pharynx. The structure and possible function of several of these muscles were previously identified and discussed by Wells (1937) and Dales (1962); however, the absence of data on different pharyngeal positions and on the three‐dimensional organisation of the musculature limited functional inferences for several structures. Paterson et al. (2014) demonstrated the value of micro‐CT for reconstructing the internal anatomy of polychaetes, particularly for the study of pharyngeal structures. Building on the work of Dales (1962), Paterson et al. (2014) applied micro‐CT to visualise and describe the pharynx of several polychaete species, including a species of *Glycera*. Although this contribution was valuable given the limited information available at the time, it did not include data on the morphology and topography of *Glycera* muscles in different pharyngeal positions, thereby limiting functional interpretations of this muscular system. Contraction of the ring muscle results in partial or complete protrusion of the pharynx (as shown in Figure 4), whereas its relaxation leads to retraction, in agreement with the description by Dales (1962). Thus, the ring muscle acts as a sphincter, preventing pharyngeal protrusion when the pharynx is not everted and allowing intermediate eversion of this structure. During pharyngeal movements, contraction and relaxation of the longitudinal muscles of the buccal tube and oesophagus may also contribute to proboscis protrusion and retraction, although Wells (1937) reported that these contractions result in only limited movement of the digestive system. These muscles are relaxed during the pharyngeal eversion, and fully contracted both when the pharynx is inverted and when it is completely everted and the jaws open. This observation suggests a possible role of these muscles in stabilising the position of the pharynx, and potentially allowing for its bending by differential stiffening and asymmetric contraction along its sides. The retensor musculature represents another structure of particular importance for pharyngeal function. In this study, six retensor muscles surrounding the pharyngeal bulb were identified. In contrast, Dales (1962) described only a single pair of retensor muscles and did not further discuss their potential functional role. Changes in retensors length observed among different pharyngeal positions, together with parallel changes in the shape of the pharyngeal bulb, suggest that these muscles may be responsible for stretching the pharyngeal bulb to its fully extended form. With regard to the intestinal retractors, 3D images of these muscles in different pharyngeal positions further clarify their potential function. Their orientation, together with their contraction during retraction, reveals a role in the controlled movement of the digestive system. As suggested by Wells (1937), the anterior intestinal retractors, which are longer and thicker, probably function as retractors of the proboscis.

A complex pharyngeal muscular system has generally been observed in Phyllodocida, although the proboscis morphology varies considerably among families and even among species within the same family or genus. For example, the hesionid genus *Oxydromus* (Hesionidae) includes both species with distinct retractor and protractor muscles allowing for the protrusion and retraction of the proboscis, and species apparently devoid of distinct retractor or protractor muscles, seemingly bearing only a series of muscles inserted in the anterior region of the buccal tube (Dales 1962). Similarly, only some Nereididae, such as *Alitta virens* (M. Sars, 1835) and *Neanthes fucata* (Savigny, 1822) are characterised by the presence of distinct posterior retractor muscles, but the majority of species apparently lack them; in this latter group, the ring muscle is expected to replace the posterior retractors in terms of function, in cooperation with the anterior retractors (Dales 1962). While this intra‐family variability in functional anatomy should be supported by further studies, possibly following the same approach of anatomical reconstruction using micro‐CT, to limit as much as possible the occurrence of artifacts linked to dissection, differences in internal anatomy and muscle development might bear some phylogenetic signal, and support the systematic revision of particularly intricate groups, such as Nereididae (Alves et al. 2023). Interestingly, the family Glyceridae is possibly also in need of systematic revision, as members of the species‐poor genera *Hemipodia* and *Glycerella* are ingroups of a large, species‐rich and evidently paraphyletic genus *Glycera*, structured in at least three clades with uncertain definition based on morphological characters (Richter et al. 2015). The available data on internal anatomy refer to only two species (*G. tridactyla* and *G. dibranchiata*) which, accordingly to the available genetic data, are closely related (Richter et al. 2015), thus they do not allow us to infer on the possible phylogenetic signal of internal anatomy in this family. Therefore, further research covering a wider array of *Glycera* species belonging to different clades could contribute to clarify this point.

Regarding the jaws, in addition to differences in jaw number and shape, there are some consistent family‐specific variations in jaw insertion and movement. For instance, in Nephtyidae the jaws are not extrudable, unlike those of Nereididae and Glyceridae (Gonçalves et al. 2023). The potential for jaw extrusion has direct bearing on the functional anatomy of Phyllodocida with armed pharynx and deserves further exploration, especially in groups less known from this point of view, such as Aphroditiformia, Goniadidae, and Hesionidae with armed pharynx. Some structures apparently similar between different families might have a completely different functionality. For instance, Nereididae also bear a ring muscle associated with the pharynx, but its contraction results in proboscis retraction in combination with the contraction of the anterior retractors (Dales 1962). In contrast, in *Glycera* specimens, contraction of the ring muscle is associated with proboscis protrusion. Nevertheless, in most Phyllodocida pharyngeal protrusion is primarily driven by coelomic hydrostatic pressure, modified by the contraction of the body wall in the midbody, while muscles associated with the pharynx are mostly involved in regulation of the pharynx movement and retraction (Dales 1962).

The eversible pharynx of Glyceridae serves multiple functions, including burrowing, feeding, sensory perception, hunting by injecting venom into its prey (Gonçalves et al. 2023), and possibly representing a defense mechanism against predators (Ockelmann and Vahl 1970). *Glycera* specimens, which prefer muddy substrates as their habitat (Klawe and Dickie 1957; Ockelmann and Vahl 1970), use their proboscis during the initial stages of burrowing, in combination with parapodial peristaltic movement (Ockelmann and Vahl 1970). Species belonging to other Phyllodocida families, such as some Nereididae and Nephtyidae, also use the proboscis for burrowing, although coelomic pulses and burial rates may differ among these taxa (Trevor 1976, 1977). According to Ockelmann and Vahl (1970), the prostomium of *Glycera* makes contact with the sediment surface and the proboscis is partially protruded, as the jaws are never fully exposed during the burrowing process. At this initial stage, the distal region of the proboscis expands and acts as an anchoring point (Ockelmann and Vahl 1970). According to the findings of the present study, the relaxation of the retensor and buccal tube longitudinal muscles is probably responsible for the expansion of the proboscis. A similar behaviour is described by Murphy and Dorgan (2011) for the glycerid *Hemipodus simplex* (Grube, 1857), but, in this case, a full eversion of the pharynx, with the opening and closing of jaws, has been observed. Afterwards, the pumping of coelomic fluid inside the pharynx, and its change in volume exert force on the crack walls to extend the burrow (Murphy and Dorgan 2011). Subsequently, the proboscis retracts, and the anterior part of the body is drawn into the newly formed tube (Ockelmann and Vahl 1970). The outcome of this burrowing behaviour is an initially vertical tube from which additional lateral tubes are created in a span ranging from hours to days (Ockelmann and Vahl 1970).

Feeding habits of *Glycera* species have been the subject of considerable debate. Although detritivorous behaviour was initially reported (Klawe and Dickie 1957; Fauchald and Jumars 1979), the presence of jaws equipped with venom glands suggests that these animals are primarily predators (Jumars et al. 2015). The jaws of *Glycera*, which are hardened by a copper‐based incrustation superimposed on a collagen‐based structure, can grab prey and inject venom through jaw openings (Gonçalves et al. 2023). *Glycera* uses its jaws to deliver venom containing a complex mixture of toxins, the composition of which is only partially understood and appears to vary substantially among species (von Reumont et al. 2014). Overall, these toxins are interpreted as a means to immobilise or injure prey organisms (D'Ambrosio et al. 2022). Among polychaetes this is a rather unique case, as production of toxins is known for a wide array of only distantly related families (D'Ambrosio et al. 2022; Righi et al. 2022; Ferri et al. 2024) but it is usually associated with epidermal structures and generally thought to function primarily in defense against predators or pathogens, rather than in prey capture (D'Ambrosio et al. 2022; Ferri et al. 2024). Significantly, in spite of previous research suggesting a role in predation of carunculine toxins produced by the fireworm *Hermodice carunculata* (Simonini et al. 2018), more recent research showed that, while the majority of the worm tissues contain detectable amounts of the toxin, they are virtually absent from the pharynx (Righi et al. 2022), which is additionally completely devoid of jaws or other offensive structures (Di Camillo et al. 2026). These recent studies support the idea that, while several polychaete groups are able to produce toxins, Glyceridae are unique in using them for predation, while even large predator species belonging to other groups were unable to repurpose defensive toxins into offensive ones. According to the micro‐CT images obtained in this study, the cushion‐like muscular swelling of the pharyngeal bulb surrounding the bases of the jaws and venom glands is most likely involved in jaw movement and envenomation. Prior to attacking, these worms remain waiting inside their tubes until they receive a mechanical stimulus that either initiates feeding behaviour or triggers withdrawal into a retreat tube (Ockelmann and Vahl 1970). Mechanoreception is also used to locate prey. Subsequent contact and testing of potential prey via mechano‐ and chemoreception (likely mediated by the pharyngeal papillae) lead either to a full predatory strike or to withdrawal of the individual (Ockelmann and Vahl 1970). While pharyngeal eversion in Glyceridae is primarily driven by coelomic fluid forced into the pharynx by contraction of the circular muscles of the metastomial segments, fine‐tuning of this process, essential for appropriate responses to stimuli and interaction with the external environment, results from a complex interplay between sensory structures (pharyngeal papillae and nerves) and accessory muscles. Our study demonstrates that this complex muscular system in *Glycera* (ring muscle, retensors, longitudinal muscles, and intestinal retractors) enables rapid and effective reactions during burrowing and feeding behaviour.

## 5 Conclusion

This study demonstrates that micro‐CT technology is a valuable tool for the advanced and detailed description of anatomical structures and the reconstruction of the muscular system of polychaetes. Such knowledge is essential for understanding the lifestyles observed in this group and their evolutionary trends (Tzetlin and Filippova 2005). Anatomical characters are closely linked to specific functions, and this relationship is fundamental for understanding the shaping of evolutionary adaptations that promote survival, energy efficiency, and resilience to environmental constraints. Studies in functional morphology demonstrate that form enables specific actions; for example, the powerful muscular bulb responsible for pharyngeal eversion in polychaetes allows rapid prey capture and efficient feeding, an adaptation that enhances energy acquisition and competitive ability (Uyeno and Kier 2015). The combination of micro‐CT with other imaging techniques (e.g. scanning electron microscopy) may further improve our understanding of proboscis functionality by enabling detailed imaging of sensory papillae and secretory structures. Furthermore, the precise delineation of muscle boundaries may be limited by the similar X‐ray attenuation of soft tissues, and complementary approaches such as histological sectioning may be required to validate specific anatomical features. Moreover, three‐dimensional reconstructions of the polychaete muscular system, together with insights into their functional roles, may be of interest in a biomimetic context, as these organisms and Annelida in general represent promising models for the development of bioinspired soft robotic systems (Filogna et al. 2023).

## Author Contributions

**Kleoniki Keklikoglou:** conceptualisation, methodology, visualisation, writing – review and editing, writing – original draft, funding acquisition. **Joachim Langeneck:** conceptualisation, writing – original draft, methodology, writing – review and editing. **Desirèe Dimichele:** methodology, writing – review and editing. **Emmanouela Vernadou:** methodology, visualisation, writing – review and editing. **Eva Chatzinikolaou:** writing – original draft, visualisation, writing – review and editing. **Luigi Musco:** conceptualisation, writing – review and editing, funding acquisition.

## Conflicts of Interest

The authors declare no conflicts of interest.

## Data Availability

The data supporting the findings of this study are available through Micro‐CTvlab (https://microct.portal.lifewatchgreece.eu/).
